# Detection of Pneumonia Associated Pathogens Using a Prototype Multiplexed Pneumonia Test in Hospitalized Patients with Severe Pneumonia

**DOI:** 10.1371/journal.pone.0110566

**Published:** 2014-11-14

**Authors:** Berit Schulte, Holm Eickmeyer, Alexandra Heininger, Stephanie Juretzek, Matthias Karrasch, Olivier Denis, Sandrine Roisin, Mathias W. Pletz, Matthias Klein, Sandra Barth, Gerd H. Lüdke, Anne Thews, Antoni Torres, Catia Cillóniz, Eberhard Straube, Ingo B. Autenrieth, Peter M. Keller

**Affiliations:** 1 Institute of Medical Microbiology and Hygiene, University of Tübingen, Tübingen, Germany; 2 German Centre for Infection Research (DZIF), partner site Tübingen, Tübingen, Germany; 3 Heart and Diabetes Center North Rhine-Westphalia, Institute for Laboratory- and Transfusion Medicine, University Hospital of the Ruhr-University Bochum, Bad Oeynhausen, Germany; 4 Heart and Diabetes Center North Rhine-Westphalia, Clinic for Thoracic and Cardiovascular Surgery, University Hospital of the Ruhr-University Bochum, Bad Oeynhausen, Germany; 5 Department of Anesthesiology and Intensive Care Medicine, Tübingen University Hospital, Tübingen, Germany; 6 University Hospital Jena, Institute of Medical Microbiology, Jena, Germany; 7 Université Libre de Bruxelles, Laboratory of Microbiology, Bruxelles, Belgium; 8 University Hospital Jena, Center for Infectious Diseases and Infection Control, and Center for Sepsis Care and Control, Jena, Germany; 9 Curetis AG, Holzgerlingen, Germany; 10 Department of Pneumology, Institut Clinic del Tórax, Hospital Clinic of Barcelona, Barcelona, Spain; 11 Institut d'Investigacions Biomèdiques August Pi i Sunyer (IDIBAPS), University of Barcelona (UB) - Ciber de Enfermedades Respiratorias (Ciberes), Barcelona, Spain; University of Delhi, India

## Abstract

Severe pneumonia remains an important cause of morbidity and mortality. Polymerase chain reaction (PCR) has been shown to be more sensitive than current standard microbiological methods – particularly in patients with prior antibiotic treatment – and therefore, may improve the accuracy of microbiological diagnosis for hospitalized patients with pneumonia. Conventional detection techniques and multiplex PCR for 14 typical bacterial pneumonia-associated pathogens were performed on respiratory samples collected from adult hospitalized patients enrolled in a prospective multi-center study. Patients were enrolled from March until September 2012. A total of 739 fresh, native samples were eligible for analysis, of which 75 were sputa, 421 aspirates, and 234 bronchial lavages. 276 pathogens were detected by microbiology for which a valid PCR result was generated (positive or negative detection result by Curetis prototype system). Among these, 120 were identified by the prototype assay, 50 pathogens were not detected. Overall performance of the prototype for pathogen identification was 70.6% sensitivity (95% confidence interval (CI) lower bound: 63.3%, upper bound: 76.9%) and 95.2% specificity (95% CI lower bound: 94.6%, upper bound: 95.7%). Based on the study results, device cut-off settings were adjusted for future series production. The overall performance with the settings of the CE series production devices was 78.7% sensitivity (95% CI lower bound: 72.1%) and 96.6% specificity (95% CI lower bound: 96.1%). Time to result was 5.2 hours (median) for the prototype test and 43.5 h for standard-of-care. The Pneumonia Application provides a rapid and moderately sensitive assay for the detection of pneumonia-causing pathogens with minimal hands-on time.

**Trial Registration:**

Deutsches Register Klinischer Studien (DRKS) DRKS00005684

## Introduction

Bacterial infection of the respiratory tract is the most common source of severe sepsis and septic shock in intensive care patients, and is one of the leading causes of death in this population. In Europe and in the U.S., the incidence of pneumonia is 1 to 5 cases per 1,000 individuals in the general population, depending on various factors like e.g. age or underlying diseases, respectively [1]–[4]. One of the hardest challenges in modern infectious disease treatment is continuously increasing resistance against anti-microbial agents resulting in frequent inappropriate empiric treatment and subsequently increased mortality [5]. The current guideline-driven strategy for empirical antimicrobial therapy in ICU patients harbours the risk for selecting antibiotic-resistant pathogens as well as being potentially insufficient for the individual patient [6]–[9]. Reasons for an inappropriate treatment may include insufficient coverage of the underlying pathogen because of primary or acquired resistance. For ventilator-associated pneumonia (VAP), the mortality rate exceeded 50% when the initial antibiotic therapy was inappropriate [10]. This number dropped to 33% when an appropriate antibiotic therapy was initially administered and was associated with a shorter duration for mechanical ventilation and a shorter ICU stay [11]. A faster diagnostic workup using molecular methods could be one option to reduce the fatal consequences of inappropriate antimicrobial therapy.

Conventional diagnostics of pathogen and resistance determination still rely on culture-based methods. However, these techniques have certain limitations (e.g. not cultivable microorganisms, decreased sensitivity in patients with prior antibiotic treatment – a frequent constellation on patients admitted to the ICU) and results are only available after one to two days after inoculation as preliminary reports, leaving correct initial antimicrobial therapy to chance. A recent study showed impressively that treatment guided by microbiological results is superior to a broad based empiric treatment in stable patients [12]. However, in instable patients guided treatment is not possible because fast point-of-care tests delivering results immediately are not yet available. Furthermore, the microbiological outcome is very sensitive to pre-analytical specimen handling and to the patient's pre-treatment with antimicrobials. Culture-independent molecular biology-based techniques such as PCR present a possibility to improve patient care. Recent studies in septic patients have demonstrated the potential power of multiplexed molecular testing approaches [13]–[15].

Herein we report the results of a clinical evaluation regarding a prototype system, a novel platform for multiplex molecular diagnostic determination of pathogens and resistance markers causing severe pneumonia - mostly bacterial infections. The objectives of this multicenter study were (1) to test a prototype of the multiplex PCR test under clinical conditions in order to adjust and validate cut-offs of this device, and (2) compare the pathogen detection performance of the device with conventional microbiological techniques in patients with suspected lower respiratory tract infection.

## Material and Methods

### Trial design

The trial was a prospective, non-interventional, non-randomized, multicenter clinical trial conducted at the following 5 European sites (in brackets: accreditation number): University Hospital Tübingen, Germany (D-ML-13130-01-00); University Hosptial Bochum/Bad Oeynhausen, Germany (DGA-ML-6638.09.02); Hôpital Erasme-Université, Brussels, Belgium (BELAC 245-MED); Hospital Clínic Villarroel, Barcelona, Spain (ER-0186/2007), and University Hospital Jena, Germany (D-ML-13144-02-00). All laboratories are certified and follow European guidelines for microbiology testing. As the test is intended to be used in critically ill patients, who have a particularly increased mortality risk in case of inappropriate treatment, only hospitalized patients were targeted.

The protocol for this trial and supporting CONSORT checklist are available as Checklist S1 and Protocol S1.

The study compared the Pneumonia Application (prototype devices, Curetis AG, Holzgerlingen, Germany) against current standard-of-care methods for pathogen detection, for 14 pathogens out of a total of 17 pathogens included in the multiplex panel: *Acinetobacter baumannii*, *Enterobacter* spp., *Escherichia coli*, *Haemophilus influenzae*, *Klebsiella oxytoca*, *Klebsiella pneumoniae*, *Moraxella catarrhalis*, *Morganella morganii*, *Proteus* spp., *Pseudomonas aeruginosa*, *Serratia marcescens*, *Staphylococcus aureus*, *Stenotrophomonas maltophilia*, and *Streptococcus pneumoniae*. For the three atypical pathogens on the multiplex panel, *Chlamydia pneumoniae*, *Legionella pneumophila*, and *Pneumocystis jirovecii*, reference tests were only done if requested by the treating physician. Data were therefore excluded from this report.

### Patient enrolment, study protocol and oversight

Samples from hospitalized adult (>18y) patients with clinical suspected pneumonia without or with antibiotic treatment were enrolled from March through September 2012. Randomly selected native respiratory samples (sputum, tracheal aspirate, bronchoalveolar lavage [BAL]) with a left-over volume of at least 1 ml were included when accepted for standard-of-care microbiology testing. Specimens were excluded in case of any of the following: Not accepted for analysis by standard-of-care, if the prototype test could not be performed on the same day as the start of microbiological testing, known tuberculosis infection, previous analysis with the prototype Application of a sample from the same patient within the past 5 days, sample type other than those mentioned above, if sample storage time has exceeded 18 hours after arrival in the laboratory.

Patient identification was removed from specimens and samples were coded (pseudonymised) and split into three aliquots prior to testing with the prototype; one aliquot was used for routine microbiology, one for testing with the prototype, and the third aliquot was stored frozen (at −20°C or colder) for discrepant result resolution done at Curetis after the end of enrolment (see below). The sampling was not trial-related and took place only when medically indicated. The prototype test was performed on the same day as the start of standard-of-care testing. Prototype test results were not used for diagnosis, treatment or other patient management decisions. Quality assurance, monitoring, and data management was conducted by a CRO (Contract Research Organization), contracted by Curetis AG, the study sponsor. Study personnel was bound to confidentiality and trained by the CRO and Curetis.

### Ethics statement

The clinical study was initially reviewed and approved by the ethics committee of the Eberhard Karls-University Tübingen, Germany (309/2011A), and afterwards by the institutional ethics committees of the other study sites, separately. The study was conducted in accordance with the Declaration of Helsinki and ICH-GCP. With the exception of the Barcelona clinic, 4 of 5 committees waived the need for informed consent as no additional patient samples were needed to perform this purely observational study. Signed written informed consent to participate in this clinical trial was obtained at the Barcelona clinic as required by Spanish law.

### Statistical methods

Sensitivity, specificity, and positive and negative predictive values were calculated by comparison of microbiological results for the 14 cultivable bacterial species to the prototype results and done by the Curetis. 95% confidence intervals were calculated according to the Wilson Score Method [16]. 'True positive' and 'true negative' (subsequently “TP” and “TN”) were defined as positive (negative) in microbiological standard method and positive (negative) in the prototype Application. Accordingly, 'false positive' and 'false negative' (subsequently “FP” and “FN”) were defined as positive (negative) in the prototype Application but negative (positive) in the standard method.

### Laboratory Methods

#### Standard-of-care methods

Standard of care microbiology was performed according to the Standard Operating Procedures (SOP) at each study site. All laboratories were quality assured according to ISO 15189. Respiratory tract samples were cultured on non-selective and selective culture media, identification of bacterial species was conducted biochemically or by mass-spectrometry. Each cultured bacterial isolate was stored at −80°C and sent to the Curetis for discrepant result resolution according to the study protocol. Microbiological results as well as patient data were transferred to an electronic case report form (eCRF) by the investigators. Investigational device raw data were sent to the CRO electronically. Non-panel pathogens were not reported from all sites consistently.

#### Prototype multiplex test

**Figure 1:**
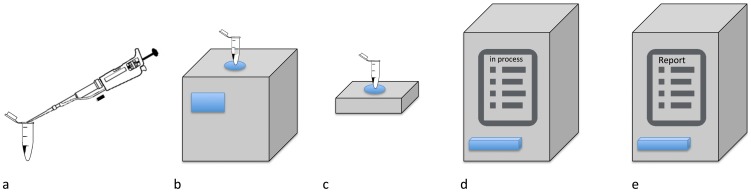
(a) Load sample tube, (b) insertion of sample tube into the Lysator, (c) transfer of sample tube and Master Mix into the Cartridge, (d) insertion of Cartridge into the Analyzer, (e) display of results.

The assay detects 16 bacterial and one fungal species known to cause pneumonia, as well as 20 genetic markers (by 22 primer pairs) coding for antibiotic resistances (not reported here). The prototype Pneumonia Application was used as recommended by the manufacturer. Briefly, 180 µl of the specimen were transferred into a sample tube. Sample lysis comprised a 30 minute protocol including mechanical, thermal, chemical and enzymatic sample treatment. The lysed sample was further processed in a prototype Cartridge. The Cartridge was pre-loaded with reagents for DNA purification, PCR primers and probes for array hybridization. The prototype Pneumonia Application integrated and automated sample lysis, genomic DNA purification, multiplex nucleic acid amplification by end-point PCR using fluorescence-labelled primers in eight independent PCR chambers with individual detection array, and qualitative amplicon detection by hybridization on a porous array membrane. The lysis protocol employed by the instrument is proprietary (patent pending). PCR and array hybridization was performed with at least three probes per analyte. A series of images of the hybridisation procedure over a specific temperature range is taken by a CCD camera. Results were derived from images processed by the proprietary software prototype. An internal control (a synthetic gene, without significant homology to known sequences) was co-processed in every PCR chamber to verify DNA purification, PCR and array hybridization. Statistical analysis of the performance data was conducted using only measurements of valid PCR chambers. Time-to-result (TTR) for the prototype test was calculated from start of the Lysator until availability of the result. Figure 1 provides an overview of the analytical procedure.

#### Discrepant Result Resolution

For all FP detections saved array images from respective prototype runs were visually screened for the presence of true positive hybridization signals. For FP detections with verified signals and for all FN detections a discrepant result resolution test was performed from the sample left-overs. In short, 180 µl of sample was treated with Proteinase K (Qiagen, Hilden, Germany) (10 min) and heated to 95°C (15 min); DNA was then isolated using the QiaAmp DNA Blood Mini Kit (Qiagen) according to the manufacturer's recommendations. DNA was amplified in single-plex PCRs with primers used in the prototype device. Amplicons were sequenced bi-directionally by a third party laboratory and identities were confirmed by “BLASTn” analysis as recommended [17]. False positive *S. pneumoniae* assay hits were confirmed by amplification of additional PCR targets against four pneumococcal marker genes (*cps*A, *lyt*A, *rpo*B, *ply*) [18]–[23].

## Results

### Patients

**Figure 2:**
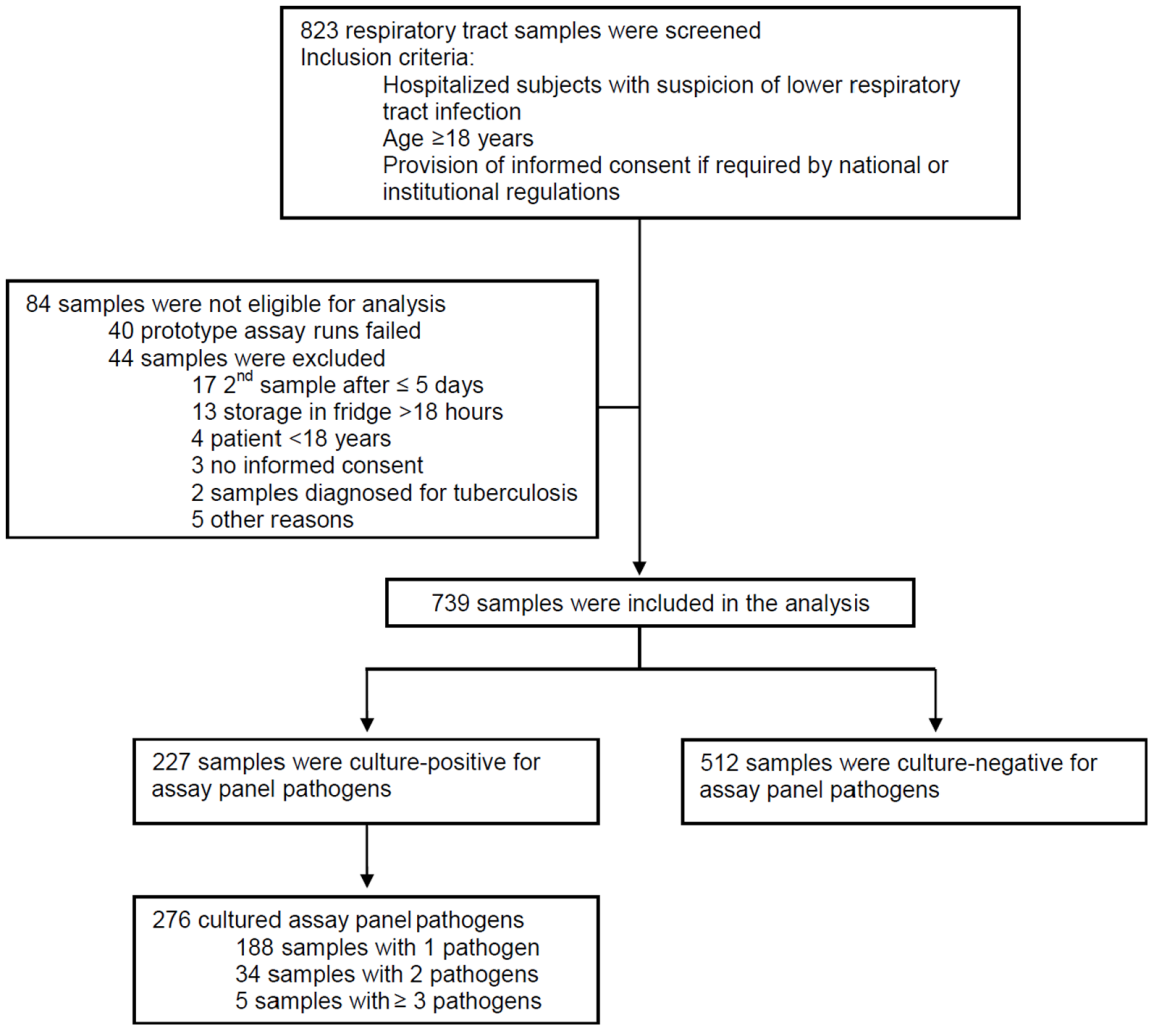
Overview on enrolment and samples for analysis.

Figure 2 provides an overview on enrolment and samples. Patient age ranged from 19 to 95 years with a median of 64 years (mean ± std. dev: 62.5±15.5 years). 523 samples were from male patients, 216 from females. The majority of samples (617 of 739) were collected from ICU patients. Of the 739 samples taken for analysis, 227 samples were positive by standard-of-care microbiology for (non-atypical) pathogens of the prototype panel (31%). In these 227 samples 276 prototype panel pathogens could be detected by culture. Additional pathogens, not covered by the prototype panel, were identified by standard-of-care microbiology, see footnote in Table 1. Results of atypical pathogens have been excluded from statistical analysis due to lack of standardisation of the reference methods at the different study sites.

**Table 1:** Pathogen performance of the multiplexed prototype assay [including discrepant results resolution in brackets].

					Sensitivity	Specificity		
Cultured organisms:	TP^a^	FN^b^	FP^c^	TN^d^	[%]	[%]	PPV^e^ [%]	NPV^f^ [%]
*Acinetobacter baumannii*	2	0	5 [1]	531	100	99.1	28.6	100
*Enterobacter spp.*	7	3 [2]	55 [14]	397	70	87.8	11.3	99.3
*Escherichia coli*	19	8 [5]	12 [7]	509	70.4	97.7	61.3	98.5
*Haemophilus influenzae*	4	0	43 [33]	473	100	91.7	8.5	100
*Klebsiella oxytoca*	0	1 [0]	5 [1]	335	0	98.5	0	99.7
*Klebsiella pneumoniae*	10	8 [7]	0	381	55.6	100	100	97.9
*Moraxella catarrhalis*	0	1 [0]	15 [12]	498	0	97.1	0	99.8
*Morganella morganii*	0	1 [0]	6 [1]	514	0	98.8	0	99.8
*Proteus spp.*	4	4 [3]	7 [0]	465	50.0	98.5	36.4	99.1
*Pseudomonas aeruginosa*	19	10 [6]	6 [3]	306	65.5	98.1	76	96.8
*Serratia marcescens*	11	1 [1]	6 [6]	529	91.7	98.9	64.7	99.8
*Staphylococcus aureus*	22	12 [11]	13 [9]	433	64.7	97.1	62.9	97.3
*Stenotrophomonas maltophilia*	20	1 [1]	46 [28]	471	95.2	91.1	30.3	99.8
*Streptococcus pneumoniae*	2	0	98 [69]*	414	100	80.9	2	100
**Total**	**120**	**50 [36]**	**317 [184]**	**6256**	**70.6**	**95.2**	**27.5**	**99.2**

a true positive: positive in microbiological standard-of-care testing and positive in the multiplexed assay.

b false negative: positive in microbiological standard-of-care testing and negative in the multiplexed assay.

c false positive: negative in microbiological standard-of-care testing and positive in the multiplexed assay.

d true negative: negative in microbiological standard-of-care testing and negative in the multiplexed assay.

e positive predictive value.

f negative predictive value [confirmed FN and FP in brackets].

* confirmation of *Streptococcus*: only as "*Streptococcus* spp."

Additional pathogens not covered by the multiplexed assay: 114 yeasts (including 85 *Candida* spp.), 7 other fungi, 9 *Citrobacter* spp., 49 coagulase neg. staphylococci, 34 enterococci, 35 streptococci (mostly *viridans* group), 8 other Gram-positive bacteria (*Leuconostoc* spp., *Rothia* spp., *Corynebacterium* spp.), 3 *Pseudomonas* spp., 10 *Neisseria* spp., 3 *Haemophilus* spp., 10 *Citrobacter* spp., 7 other Gram-negative bacteria (*Ralstonia* spp., *Achromobacter* spp., *Burkholderia* spp., *Raoultella* spp., *Serratia* spp.).

### Device Performance

Analysis of the prototype run validity, corresponding to results from the eight PCR chambers in each cartridge, yielded 65.7% valid measurements. The reasons identified for invalid results within successful runs were grid failures (4.9%), pumping failures (5.6%), and control gene failures (23.8%). ‘Grid failures’ occured when the hybridization array was not correctly identified by the detection software. ‘Pumping failures’ were either caused by insufficient washing during hybridization or inadequate buffer distribution to PCR chambers. ‘Control gene failures’ were caused by either failure to obtain a DNA eluate, PCR failure or by fluidic failures. TTR for the prototype test yielded a median of 5.2 hours (1^st^ and 3^rd^ quartile 5.1 and 5.3 hours). The TTR median for standard-of-care was 43.5 h (1^st^ and 3^rd^ quartile 25.2 and 70.1 hours) for pathogen identification.

Valid chamber results were generated for 170 of the 276 pathogens detected by microbiology (positive or negative detection result by multiplex prototype assay). Among these, 120 pathogens were identified by the prototype device, 50 pathogens were not detected (Table 1). Overall performance for pathogen identification was 70.6% sensitivity (95% CI lower bound: 63.3%, upper bound: 76.9%, and 95.2% specificity (95% CI lower bound: 94.6%, upper bound: 95.7%). As shown in Table 1 sensitivity strongly depends on the bacterial species. Notably, while the PPV varied between 2.0% for *S. pneumoniae* and 100% for *K. pneumoniae* the NPV reached >96% for all pathogens of the panel within the study population of the five study sites.

Discrepant (FP and FN) results were resolved as described in Material and Methods. 50 pathogens (FN) were not detected by the prototype of which 36 were confirmed by subsequent positive PCR/sequencing results as false negative. 14 pathogens could not be detected in the manual retesting.

Vice versa, the prototype assay detected additional 317 pathogens, of which 184 were confirmed by PCR/sequencing demonstrating their presence in the sample. Of 133 non-confirmed detections, 48 corresponded to array image artefacts (either caused by insufficient washing, or particles on the membrane or software analysis errors). In one case the initially identified *Enterobacter* spp. could not be confirmed by the manual PCR/sequencing procedure. BLASTn analysis revealed the presence of *K. pneumoniae* DNA in the sample indicating a cross reaction the primer pair used in the assay.

Most remarkable was the high number of false positive *S. pneumoniae* detections (N = 98) of which 29 were not reproducible by single-plex PCR. The remaining 69 cases could not be clearly assigned to specific streptococcal species by BLASTn analysis of sequenced PCR products due to insufficient database coverage and variability of the sequenced DNA fragment, but showed similarities to the members of to the *Streptococcus mitis* group. For further analysis of this special issue 4 additional PCR targets demonstrating pneumococcal marker genes (*cps*A, *lyt*A, *ply*A, *rpo*B) were chosen and amplified. Finally, 5 of 69 initial detections were confirmed by a positive result in all 4 additional PCRs as “*S. pneumoniae*”.

The results from this study with the prototype device were taken to improve the performance of the next generation of the Pneumonia Application by the company. In detail, the detection of washing failures, the adaptation of melting temperature ranges, and cut-off changes to optimize sensitivity and specificity were adjusted. Changed parameters were collected and validated using the pool of the described study data. Raw data were stored as series of images from each reaction chamber of each run, thus it was possible to re-analyze and re-calculate the complete data pool using the new settings without changing the obtained original data. Both overall sensitivity and specificity were increased after re-calculation (sensitivity: 78.7%; 95% CI lower bound of 72.1%, upper bound: 84.0%, and specificity: 96.6%; 95% CI lower bound of 96.1%, upper bound: 97.0%) (data not shown).

Furthermore, re-testing of 123 study sample left-overs on commercial CE-marked Unyvero devices with improved settings confirmed the results obtained by re-calculation of the study data pool (data not shown).

## Conclusions

Pneumonia Application testing of respiratory samples is a rapid approach to detect clinically relevant pneumonia-causing pathogens in a fully automated manner. The panel of detectable pathogens was chosen according to pathogens relevance in multicenter studies on pneumonia and after expert consultations [24]. As demonstrated in the European multicenter study, sensitivity for in-panel organisms varies greatly in the prototype devices, reaching 100% for 4/17 pathogens and 70.6% overall.

The prototype device detected 184 additional pathogens ( =  confirmed FP) in the study samples after discrepant results resolution in comparison to standard-of-care methods. Samples included in the study were mainly (83.5%) collected from ICU patients due to the study centers being tertiary care teaching hospitals with a case-mix of multi-morbid patients and solid-organ transplant recipients. As expected, more gram-negative *Enterobacteriaceae* such as *E. coli* and non-fermenters such as *P. aeruginosa* were detected in our study patient population than in a setting with non-pretreated patients with community-acquired pneumonia. According to our study data, a high proportion of the patients were pretreated with antibiotics (pre-treated 47,8% of patients, not pre-treated 5,3%, unknown 47,0%), which may in part explain the discrepancy between pathogens detected by the prototype but not found in culture. The high number of “false positive” for streptococci, *H. influenzae*, and *M. catarrhalis* could reflect an asymptomatic carriage with a normal oro-pharyngeal flora, which was not reported by the standard culture. Another explanation would be amplification of DNA of dead microbial organisms, which were not relevant for the patient's course of disease. Extensive discrepant results resolution enabled us to gain insight into the potential causes: In a re-sequencing analysis applying in-test PCR primers and conditions demonstrated that 69/98 samples with positive *S. pneumoniae* test result contained streptococcal DNA of either *S. pneumoniae* (of which 5 could be confirmed by additional PCR targets) or non *S. pneumoniae* streptococci. Using BLASTn analyses on the GenBank database amplicon sequences of the primary PCR allowed no distinction of *S. pneumoniae* sequence-type and nearly related *S. mitis* group sequences. According to previous studies, molecular detection and identification of *S. pneumoniae* is challenging because neither a single PCR target (e.g. *pylA*) is present in all strains nor is a single target specific for *S. pneumoniae* (e.g. 16S rRNA gene, *rrs*) [25]. On the other hand differentiation of *S. mitis* group streptococci also causes problems in clinical microbiology laboratories, which could explain the microbiology confirmed *S. pneumoniae* showing only weak signals with the comparative *S. pneumoniae* PCRs.

Nucleic acid amplification techniques cannot differentiate between living and dead organisms. This might explain a proportion of positive detection results in the PCR test in comparison to cultivation-based techniques. Previous studies examining blood-stream infections by PCR methods allowed detection of bacterial DNA up to 60 days after initiation of antimicrobial therapy [26]. Persistence of amplifiable microbial DNA in respiratory samples of pre-treated pneumonia patients has not been examined in previous studies for all in-panel organisms.

After data re-analysis using production-device settings only 38 false-negative PCR results were generated for in-panel organisms. When comparing our data to previous multiplex PCR test related studies for respiratory samples, comparable sensitivity and specificity results were obtained for bacterial pathogens [27], [28]. Negative predictive values between 98.2 and 100% illustrate the strength of the test to confirm absence of in-test organisms.

In summary, the Curetis Pneumonia Application is the first fully-automated multiplex PCR-based diagnostic device entering the market. We have assessed performance of a prototype in a prospective multi-center study using routine respiratory samples. The assay has several critical advantages over conventional nucleic acid-amplification tests, which have been licensed in the last 20 years. The Pneumonia Application is simple to perform, is not prone to cross-contamination, requires minimal biosafety facilities and has a moderate to high sensitivity of up to 100% for in-panel organisms. However, we could demonstrate that the prototype devices have specificity issues regarding *Enterobacter* spp., *H. influenzae* and *S. pneumoniae* in comparison to culture-based methods. Specificities for all 3 organisms were distinctly increased with the cut-off settings of the series production devices. Although 739 patient samples have been measured throughout the study, insufficient case numbers have been obtained for statistical analysis of the 22 resistance genes on the panel. Due to the use of prototype instruments in the study and the manual manufacturing of the consumables, a significant rate of invalid test runs has occurred. Test turn-around times for the instrumentation were remarkably short. This is adding much to the impact of microbial testing for clinical treatment decisions. Routine microbial testing in community-acquired pneumonia patients is not yet recommended by guidelines as standard of care particularly for out- patients mainly due to long test turn-around times [29]. For further application, the study allowed recalculation of detection signal limits and revision of the software interpretation algorithms already improving sensitivity and specificity values in an *in silico* re-analysis of the study raw data.

Early detection of additional causative pathogens by a sensitive PCR-based method has the potential to reduce the proportion of patients with initial inappropriate treatment [5]. In contrast, detection of non-causative microorganisms may promote antibiotic overuse. Clinical relevance depends on detected concentrations and origin of specimens, e.g sputum or lavage. Furthermore, some microorganisms may be part of the normal flora of healthy individuals, like e.g. streptococci or *H. influenzae*, whereas others are regarded as disease relevant, even when present in trace amounts.

The investigational system is intended to support treatment of severely ill patients where rapid appropriate treatment instead of empirical antibiotic regimens is absolutely essential. For these patient cohorts, the benefits of rapid and sensitive detection will presumably overweigh disadvantages of potential antibiotic overuse. PCR-based results together with other diagnostic data and the clinical appearance of the patient will support the physician to define optimal treatments much more rapidly than by conventional methods alone. A full-cost calculation covering standard microbiological workup including microscopy and culture in comparison to molecular testing cannot be given, as the device under examination was a prototype instrument without given price list for consumables. We expect, that the molecular test in the final product may be more costly as culture based methods.

The potential improvement of care by such a system lies primarily in the early detection of pathogens that are not covered by empiric treatment recommended in guidelines. However, clinical benefit of such a new method needs to be demonstrated in additional studies.

## Supporting Information

Checklist S1 CONSORT 2010 checklist of information to include when reporting a randomised trial.
(DOC)

Flow Diagram S1 CONSORT 2010 Flow Diagram.
(DOC)

Protocol S1 Study Protocol.
(PDF)

Study Registration S1 Application for registration for trial DRKS00005684.
(PDF)
