# Supplementary material for: Detection of Pneumonia Associated Pathogens Using a Prototype Multiplexed Pneumonia Test in Hospitalized Patients with Severe Pneumonia
Source: PLoS One. 2014 Nov 14;9(11):e110566. doi: 10.1371/journal.pone.0110566 (PMC4232251; doi:10.1371/journal.pone.0110566)
Supplement: Flow Diagram S1 — CONSORT 2010 Flow Diagram. (DOC) [file pone.0110566.s002.doc]

**CONSORT 2010 Flow Diagram**

**Analysis**

**Enrollment**

Assessed for eligibility (n=823)

Excluded (n=84)

♦  Not meeting inclusion criteria (n=39)

♦  Declined to participate (n=3)

♦  Other reasons (n=42)

Analysed (n=739)
♦ Excluded from analysis (give reasons) (n=0)

Included (n=739)
