# Supplementary material for: Detection of Pneumonia Associated Pathogens Using a Prototype Multiplexed Pneumonia Test in Hospitalized Patients with Severe Pneumonia
Source: PLoS One. 2014 Nov 14;9(11):e110566. doi: 10.1371/journal.pone.0110566 (PMC4232251; doi:10.1371/journal.pone.0110566)
Supplement: Protocol S1 — Study Protocol. (PDF) [file pone.0110566.s003.pdf]

curetis

Curetis Study Protocol

Study Title

Detection of Pneumonia associated Pathogens and Antibiotic Resistance Genes using the Curetis Unyvero® Pneumonia Application

Introduction 6
Clinical Trial Design 8
Study Preparation 11
Study Procedure 12
Adverse Events and Effects 16
Statistical Methods 17
Discrepant Result Analysis 19
Appendix 20

Description

This document describes the clinical study (Unyvero Pneumonia Performance, code CS-2011) for the investigational device “Unyvero Pneumonia Application”.

Confidential

This investigational plan, its contents, and the information relating to it are the property of Curetis AG. All information is to be kept confidential.

© Copyright 2011 Curetis AG - Confidential

Coordinating Investigator

Sign:______________________________

Date:______________________________

-

Biostatistician

Prof. Dr. Dr. Timm
Kompetenzzentrum für klinische Studien
Bremen
Achterstrasse 30
28359 Bremen, Germany

Sign:______________________________

Date:______________________________

Sponsor Personnel

Sign:______________________________

Date:______________________________

Sign:______________________________

Date:______________________________

Study Management

Dr. Andreas Grund, PhD
Managing Director/ CEO
GCP-Service International Ltd. & Co.KG
Anne-Conway-Str. 2
28359 Bremen
Germany

Sign:______________________________

Date:______________________________

© Copyright 2011 Curetis AG - Confidential

Investigator Statement

I agree to perform this trial, to maintain the procedures required to carry it out and to abide by the terms of this protocol. This clinical trial protocol is confidential and the property of Curetis AG and will not be used, disclosed or published without its written consent.

Investigator Signature

Name: ______________________________

Title: ______________________________

Address details: ______________________________

____

____

Signature: ______________________________

Date: ______________________________

© Copyright 2011 Curetis AG - Confidential

Introduction

Background

Pneumonia is one of the most common infectious diseases worldwide, usually caused by a bacterial infection. In Europe and in the US the incidence is 1 to 10 cases per 10,000 inhabitants, depending on various factors as e.g. age. For Germany there are about 200,000 hospital acquired cases per year and some 400,000 community acquired cases per year with more than 10,000 cases of death (Lorenz et al., 2003; Höffken et al., 2009).

The standard method for Pneumonia causing pathogen identification is microbiology culture diagnostics. However, the results are not available until 2 to 5 days after sampling, and detection rates for pathogens are well below 100 percent. Thus, initial therapy is mostly empirical and often uses broad spectrum antibiotics until microbiology results are available. As a consequence, initial therapy is often inadequate, thereby extending the disease period and hospital stays. According to studies, 20% to 50% of pneumonia cases are initially not treated adequately (Rello et al. 1997; and personal communications). In intensive care units, the mortality rate in pneumonia cases ranges from 20% to 30% with initially adequate therapy to 50% to 80% when initially not treated adequately, i.e. when antibiotic regimen was changed after microbiology results were available (Luna et al. 1997, Kollef et al. 1999). Furthermore, inadequate / unspecific antibiotic therapy accelerates the spread of resistant pathogens. This underlines the strong need for fast and precise diagnostics.

The Curetis Unyvero Pneumonia Application is intended to aid in the diagnosis of pneumonia and identifies genes of 17 pathogens and 22 genes associated with antibiotic resistances in less than 4 hours.

In the present study, the performance of the Unyvero Pneumonia Application shall be tested under clinical conditions and compared to microbiology standard-of-care, including comparison of the test results and the time to result. The study design prevents any potential risk for the patient because (a) only left-overs from clinically indicated respiratory samples are used, (b) the test results are not made available to the treating physician at any time, and (c) the study is not interventional.

Scope

This protocol is valid for Curetis' first product, the 'Unyvero Pneumonia Application' for the detection of pathogens and their antibiotic resistance genes from human respiratory samples of patients suspected with pneumonia.

© Copyright 2011 Curetis AG - Confidential

Product Description

The investigational medical device 'Unyvero Pneumonia Application' is a fully automated and integrated device intended for aiding in the diagnosis of pneumonia from a patient sample through simultaneous detection of pathogens and their antibiotic resistance genes. The 17 pathogens identified using the Curetis Unyvero Pneumonia Application are summarized in Table 1.

Acinetobacter baumannii	*Moraxella catarrhalis*
Enterobacter spec.	*Pneumocystis jirovecii*
Escherichia coli	Proteus spec.
Chlamydophila pneumoniae	*Pseudomonas aeruginosa*
Haemophilus influenzae	*Serratia marcescens*
Legionella pneumophila	*Staphylococcus aureus*
Klebsiella pneumoniae	*Streptococcus pneumoniae*
Klebsiella oxytoca	*Stenotrophomonas maltophilia*
Morganella morganii	

Table 1: Bacteria tested by the Unyvero Pneumonia Application

The device identifies the resistance markers: tem, shv, ctx-M, dha, ebc, kpc, oxa 51, mecA, msrA, ermA, ermB, ermC, mef A/E, int1, sul1, gyrA83, gyrA87 and parC, encoded by 22 genes sequences. Refer to the Instructions for Use for more information.

Intended Use/Indications for Use

The current proposal for the Intended Use Statement can be summarized as follows:

The Curetis Unyvero Pneumonia Application is a qualitative nucleic acid multiplex test intended for the simultaneous detection and identification of multiple pathogen-derived nucleic acids in sputa, respiratory aspirates and bronchial lavages from individuals suspected of pneumonia, in order to provide information on pathogen species and antibiotic resistance genes. The detection and identification of specific non-viral pathogen-derived DNA from individuals exhibiting signs and symptoms of pneumonia aids in the diagnosis of respiratory bacterial or fungal infection if used in conjunction with other clinical and laboratory findings.

© Copyright 2011 Curetis AG - Confidential

The following pathogen and resistance markers are identified using the Curetis Unyvero Pneumonia Assay in a qualitative way: final panel with Bacteria and Resistance genes will be defined after clinical study. Concomitant cultures are necessary to recover targeted organisms for further susceptibility testing.

It is recommended that specimens found to be negative for pathogens and resistance genes after examination using the Curetis Unyvero Pneumonia Application are confirmed by microbiological culture. Especially, negative resistance results do not preclude lack of this resistance, as other molecular mechanisms not tested for by the Unyvero Pneumonia Application could still contribute to such a resistance phenotype, and should not be used as the sole basis for diagnosis, treatment or other management decisions. Positive results do not rule out fungal or viral co-infection, or co-infection with other bacteria not present on the Unyvero Pneumonia panel. The agent detected may not be the definite cause of disease. The use of additional laboratory testing (e.g. bacterial culture) and clinical presentation must be taken into consideration in order to obtain the final diagnosis of pneumonia.

Clinical Trial Design

This is a non-interventional, controlled, open, non-randomized, multicenter clinical study that compares current standard-of-care for pathogen and antibiotic resistance detection (i.e. culture) to a new diagnostic device, the Unyvero Pneumonia Application (based on molecular diagnostic methods).

The study will use respiratory specimens taken for standard-of-care (i.e. microbiology testing) from hospitalized subjects suspected with lower respiratory tract infections. Specimens will be split into three aliquots; one aliquot will be used for standard diagnostic testing, one for investigational testing and the third aliquot will be stored for discrepant result resolution. The sampling is not trial-related and takes place if and when medically indicated.

As the device is under investigation, the test results provided by the Unyvero Pneumonia Application will not be made available to the treating physician and therefore will not be used for diagnosis, treatment or other management decisions.

For sample processing in the study only a sample ID will be used so that patient identification by the sponsor will not be possible.

For data protection reasons, the investigational device will not be connected to any LIS or HIS. The analysis of the samples will not be used for human DNA analysis and samples will not be kept for more than five years.

© Copyright 2011 Curetis AG - Confidential

Study Objectives

Primary Objective

The primary objective of this clinical trial is to evaluate the sensitivity for each pathogen covered by the panel for the investigational IVD.

Secondary Objectives

In addition, further objectives are

1. to evaluate the specificity of species detection by the investigational diagnostic test,
2. to correlate genotypic resistance detection results of the investigational diagnostic test with the phenotypic resistance determination by standard procedures.
3. to compare the 'time to result' of each pathogen and resistance gene detected by the investigational diagnostic test to standard procedures.
4. to compare discrepant results of the investigational diagnostic test to the standard-of-care.

Study Endpoints

Primary Endpoint

Determination of sensitivities of pathogen detection by the investigational test for each pathogen listed in the test panel.

Secondary Endpoints

1. Evaluation of specificity of pathogen detection by the investigational test for each pathogen listed in the test panel.
2. Evaluation of correlation between results of genotypic resistance detection by the investigational test and phenotypic resistance detection by standard-of-care.
3. Comparison of "time to result" of each pathogen and resistance gene detected by the investigational diagnostic test with standard procedures.
4. Evaluation of discrepant results of the investigational diagnostic procedure, following discrepant result resolution concerning pathogen detection.

Definitions

Sensitivity for pathogen detection will be determined in all samples in which standard-of-care have identified pathogens that are covered by the panel of the investigational IVD. Sensitivity is defined as:

$$Sensitivity = \frac{number\ of\ true\ positives}{number\ of\ true\ positives + number\ of\ false\ negatives}$$

Specificity for pathogen detection will be determined for all samples included in the trial. Individual specificities will be calculated for the detection of the respiratory pathogens analyzed by the investigational IVD compared to the standard-of-care. Specificity is defined as:

© Copyright 2011 Curetis AG - Confidential

$$Specificity=\frac{number\ of\ true\ negatives}{number\ of\ true\ negatives+number\ of\ \ false\ positives}$$

“Time to result” is measured as time from “sample splitting” to final finding (“ready to report”) of each pathogen and resistance gene covered by the panel of the investigational IVD, and antibiotic resistance as measured by standard procedures.

Discrepant results of pathogen detection will be evaluated in all samples with discrepant results of the investigational IVD compared to standard methods, except for those samples diagnosed for tuberculosis. Specificity will also be calculated comparing against the combined result of standard-of-care and bi-directional sequencing.

Duration of the Investigation

Duration of the trial at each of the participating sites is estimated to be approximately 6 months from inclusion of first subject to the day of final analysis of the last subject’s sample. The trial will be divided into two parts, with a first part conducted in about 4 centers in Europe with an estimated duration of 8 months, and a second part conducted in about 3 to 4 centers in the USA with an estimated duration of 8 months.

Subjects

Clinical Trial Population

Hospitalized male and female individuals with suspicion of lower respiratory tract infection. The study shall include a minimum of N =2,000 subjects with up to 1,000 subjects in Europe and at least 1,000 subjects in the US. It is intended to achieve balanced numbers of samples over all study sites. Subjects participate in the clinical trial for sampling only.

Indication

Suspicion of pneumonia

Inclusion Criteria

- Hospitalized subjects with suspicion of lower respiratory tract infection.
- Age at least 18 years.
- Any of the following sample types: sputum, respiratory aspirate, bronchial lavage, combi cath®, and protected brush~~and bronchial lavage~~ taken for standard-of-care.
- Left-over sample amount ≥ 1 ml (in addition to the volume required for standard procedures).
- If required by national or institutional regulations, signed written informed consent to participate in this clinical trial, with the exception for unconscious patients. For these patients the written informed consent has to be obtained as soon as subjects are conscious or from a person authorized to give informed consent for this subject.

© Copyright 2011 Curetis AG - Confidential

Exclusion Criteria

- Sample is not accepted for analysis by standard-of-care.
- Samples for which Curetis test cannot be performed at the same day of start of microbiology testing.
- Known infection with tuberculosis
- Out-patient or ambulant patient.
- Previous analysis of a respiratory sample with the Unyvero Application from the same patient within the past 5 days.
- Patient known to have been enrolled in another clinical study within the last 6 months.
- Other sample types, like nasal swab or brush, bronchial swab or brush, blood, throat washing, drainage, gastric juice, pleural punctuates.
- After arrival in the laboratory, sample storage time has exceeded up to 18 hours in the lab refrigerator, including up to 2 hours at room temperature (18 hours in total).

Study Preparation

Initia~~liza~~tion Visit

During this visit standard-of-care of the following procedures will be recorded for each study center:

- SOPs for microbiology testing for each pathogen and antibiotic resistance part of the Unyvero panel (i.e. type of agar or antibiotics used, as well as testing technologies used for non-cultured pathogens).
- SOPs for quality control measures for these testings.
- SOPs for sampling and sample qualification for microbiology.

The clinical monitor will ask for documentation of quality protocol measures for all standard procedures and documents where the Unyvero Pneumonia Application will be performed.

Required Devices and Materials

The sponsor provides one Unyvero Lysator, one Unyvero Analyzer, a computer with touchscreen monitor, a printer (if required), a bar code reader and software. Secondly the sponsor delivers the required number of Unyvero Pneumonia Cartridges, Unyvero Pneumonia Sample Tubes with Caps, Unyvero Master Mix Tubes, Test Samples and Unyvero Transfer Tools. In addition, the adequate number of disposables for training and precision studies will be shipped prior to study initiation. The provided positive and negative Test Samples contain analytes from the Unyvero Pneumonia panel, respectively do not contain any panel analyte.

Prior to first subject in familiarization runs and a precision and reproducibility study have to be conducted.

© Copyright 2011 Curetis AG - Confidential

Familiarization/Training

In order for the personnel to become familiar with the Unyvero work flow, a series of at least 3 Test Samples shall be run by every person who will run tests as part of the clinical study. The results of this training shall be sent to the Sponsor immediately after completion by email for evaluation. The precision/reproducibility program will not be started before positive feedback from sponsor on the familiarization outcome.

Precision and Reproducibility Study

In-depth precision and reproducibility studies are part of Curetis pre-clinical testing. However, to ensure a good comparison of the clinical data all study sites will conduct precision / reproducibility studies with Test Samples analyzed as summarized in Table 2. For details see the SOP 'Unyvero Reproducibility Study'.

Day	Minimum required analysis of Test Samples		Min. total number of Test Samples per site
	Positive Test Sample	Negative Test Sample	
1	2	1	15
2	2	1	
3	2	1	
4	2	1	
5	2	1	

Table 2: Overview Test Samples for Precision and Reproducibility Studies

The results of the reproducibility runs shall be stored electronically and on paper and immediately be sent to Curetis for evaluation. The clinical study will not be started before positive feedback from sponsor was obtained on the outcome of this precision and reproducibility tests.

Study Procedure

Figure 1 illustrates the study work flow. When the sample has arrived in the microbiology laboratory and has qualified for enrollment, its left-over from standard-of-care testing will be used for

1. analysis with the investigational device
2. storage for later discrepant result resolution.

The microbiology testing is done as requested by the physician and as routinely performed in the laboratory. For each site the standard microbiology testing procedures will be recorded in the Initia~~liza~~tion Visit Protocol. Part of the left-over sample will be processed with the Unyvero Pneumonia Application as described

© Copyright 2011 Curetis AG - Confidential

in the instructions for use. The other part will be stored (see for details below) and shipped to Curetis at a later point in time to serve for further investigations performed by Curetis AG. In addition, isolates will be collected from samples with positive microbiology results.

After finalization of each individual subject's enrollment, the respective results of standard microbiology testing will be compared to Unyvero results. In case of discrepant results additional testings will be arranged for by Curetis AG for pathogen and antibiotic resistance gene detection as described in the SOP 'Discrepant Result Resolution'.

The description of the standard-of-care quality control measures used for microbiology testing will be recorded in the Initia~~liza~~tion Visit Protocol. The investigational device will be monitored

- systematically during each run by an internal positive control
- in regular intervals with two different Test Samples according to the SOP 'Quality Control Runs'.

Standard safety procedures as required by country or local regulations regarding the handling of infectious materials should be followed, please refer to Instructions for Use.

All study data will be recorded on Case Report Forms (CRF) or electronic Case Report Forms (eCRF).

© Copyright 2011 Curetis AG - Confidential

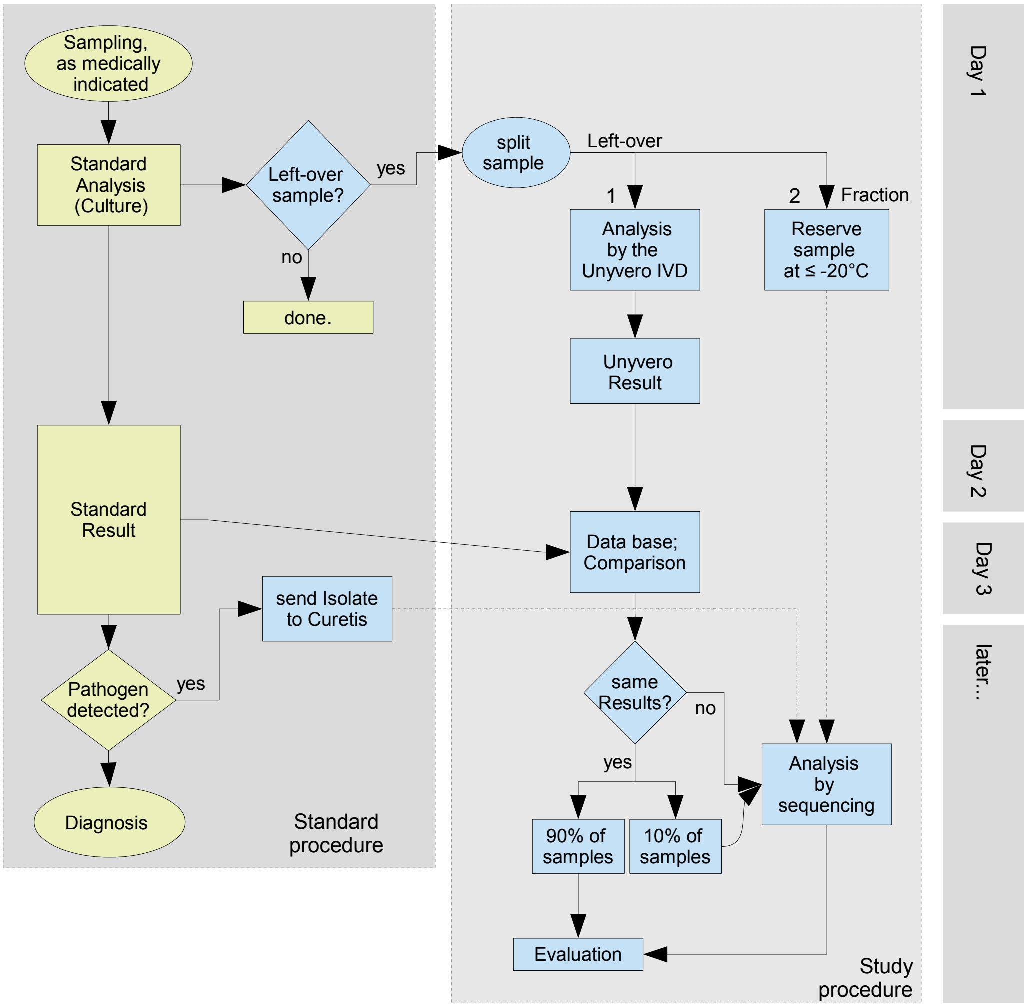

Figure 1: **Study Work Flow**

Sample Blinding

Left-over samples will be blinded so that subject identification is not possible. Blinding will not be done by the same person that performs the subsequent analysis with the Unyvero system.

The patient identification will be removed from the specimen and a sample number is assigned to each study sample according to the following scheme: C_Ce_N where C identifies the country by country telephone code. Ce identifies the study center and N identifies the sample. The number N will be given to each~~. Ce identifies the study center and N identifies the sample. The number N will be given to every~~ sample at each site. Example: 49_1_022 refers to sample no. 22 from center 1 in Germany. The code is illustrated in table 3.

Site	Number C_Ce
Europe, site 1	49_1
Europe, site 2	34_1
Europe, site 3	49_2

© Copyright 2011 Curetis AG - Confidential

Europe, site 4	49_3
US, site 1	01_11
US, site 2	01_12
US, site 3	01_13
US, site 4	01_14

Table 3: Coding of Study Sites

Clinical Trial Work Flow

Subjects attend only normal clinic routine visits. Since this study uses only left-over specimens and the study is non-interventional, there will be no trial-related visits for the subjects.

Date 1 (day 1)

- Follow standard safety procedures as required by country or local regulations regarding the handling of infectious materials.
- Use sampling procedure according to standard-of-care in each site and as documented in Initia~~liza~~tion Visit Protocol. Record date and time of sampling.
- Enroll only subjects
  - which are hospitalized
  - which were not enrolled in the study within the past 5 days
  - which are not known to have been enrolled in another study in the past 6 months.
  - whose samples were qualified for microbiology testing (e.g. < 10 epithelial cells per ml bronchial lavage), for details refer SOP 'Sample Qualification'
  - whose samples have a left-over volume after standard-of-care testing of at least 1 ml, allowing two additional fractions of 0.5 ml each
  - if required by national or institutional regulations,if informed consent is available with the exception for unconscious patients. For these patients the written informed consent has to be obtained as soon as subjects are conscious or from a person authorized to give informed consent for this subject.
- Record subject age, gender and clinical setting (i.e. ICU, ward).
- After arrival, sample should been processed within 2 hours[1]
- Analyze the specimen according to standard procedures. Record start time of standard analysis.
- Split left-over in the microbiology laboratory without any pre-processing in two aliquots (1 and 2). Record date and time of sample splitting.
- If stored, record storage conditions and time.
- Label both fractions with the same sample number

1 If the sample cannot be processed as required , the sample will be stored at 4°C until processing for up to 16 hours (18 hours in total).

© Copyright 2011 Curetis AG - Confidential

- Use the first fraction of the sample (0.5 ml) as follows:
  - Use 200 µl for the analysis with the investigational device as described in the Instructions for use
  - Store the remaining raw sample (300 µl) immediately at 4°C for optional repetition of the investigational test if necessary, i.e. in the case of an invalid run.
- Store the second fraction immediately at at least -20° C for later shipment to Curetis.
- In case of an invalid run with the Unyvero Pneumonia Application (i.e. instrument failure) repeat the test using the remaining raw sample stored at 4°C at the same day.
- Record the final findings (detection and non-detection, "ready to report") of the investigational IVD. The findings of the investigational IVD shall not be reported to the clinician as results from the Unyvero system shall not be used to make any therapy decisions.

Subsequent Dates

- Document the following clinical data as of date of sample taking as asked in the CRF:
  - ventilation (yes/no/unknown)
  - x ray (yes/no/unknown, result)
  - immune suppression as defined in CRF (yes/no/unknown)
  - body temperature >38°C (yes/no/unknown, since)
  - type and dosage of antibiotics within the last 48 hours before sampling and at the time of sampling.
- Follow-up microbiology testing until final results are available,
- Record all findings of standard-of -care (including gram-stain) as asked the CRF / eCRF (detection and non-detection, "ready to report") for pathogens and resistances with date and time, when the result was available.
- Record all microorganisms (including host flora) and resistances that are identified.
- For all microbiology positive samples collect isolates of all pathogens, handle those as described in the SOP 'Handling of Isolates' and store those at at least -20°C for later shipment to Curetis AG.
- Ship aliquot 2 and isolates to Curetis AG on dry ice for discrepant result resolution, when requested by Curetis staff. Samples diagnosed for tuberculosis will be excluded from discrepant result resolution and will not be shipped.

Adverse Events and Effects

For medical device studies, an adverse event is defined according to the

© Copyright 2011 Curetis AG - Confidential

Standard ISO 14155:2011 “Clinical investigation of medical devices for human subjects -- Good clinical practice” as „any untoward medical occurrence in a subject“. An adverse event related to a device is defined as an „adverse device effect“ and is defined as „any untoward and unintended response to a medical device“.

A serious adverse event is defined as an adverse event that:

- led to death.
- led to a serious deterioration in the health of the patient that resulted in a life-threatening illness or injury,
- resulted in a permanent impairment of a body structure or a body function
    - required in-patient hospitalization or prolongation of existing hospitalization,
    - resulted in medical or surgical intervention to prevent permanent impairment to body structure or a body function
    - led to fetal distress, fetal death or a congenital abnormality or birth defect.

However ISO14155 is not applicable for this investigation due to the “low risk” character of the device. Due to the study design the investigational device does not come into contact with study subjects and the results of the analyzed study data will not be made available to the investigators so that the possibility of harming a study subject does not exist. This makes the occurrence of ADEs or SADEs extremely unlikely. Nevertheless in order to ensure health and welfare of study subjects and users all UADEs will be reported in the CRFs and reported within 48 hours to the sponsor in case of a serious UADE

Statistical Methods

Cohort Size Estimation

Based on literature search average incidences for each pathogen causing bacterial pneumonia were calculated (see table 4 in Appendix). As the study intents to claim a lower bound of 90% of the 95% confidence interval for sensitivity, it will be required to measure 73 positive samples of each pathogen (see table 4, column “number of positive samples” =73).

As indicated in table 4, the prevalence of some pathogens is below 6 % in pneumonia patients. Therefore, insufficient numbers may be collected during the study period for some of these pathogens. For these pathogens a lower bound of 80% of the 95% confidence interval will be anticipated and retrospective sampling may be considered. The required sample number for a lower bound of 80% of the 95% confidence interval is 8 positive samples.

Taking into account the prevalence of each pathogen, a total of 1,300 positive samples should be analyzed: Assuming a ‘microbiology positive rate’ of 66% for

© Copyright 2011 Curetis AG - Confidential

respiratory samples, the study should include at least 2,000 subjects (i.e. about 1,300/0.66), also covering invalid measurements.

Statistical Analysis

Analysis will be performed on only those samples for which both an investigational test result and a standard procedure result with completed CRF are available. Data shall be discarded if the inclusion criteria are not met, if the protocol has not been followed, or if the result cannot be correctly allocated to the sample (i.e. due to incorrect sample labeling).

Sensitivity for pathogen detection will be determined for all samples in which standard procedures have identified pathogens that are covered by the panel of the investigational IVD. Individual sensitivities will be calculated for the detection of the respiratory pathogens analyzed by the investigational IVD compared to the standard procedures. Sensitivity is defined as:

$$Sensitivity=\frac{number\ of\ true\ positives}{number\ of\ true\ positives+number\ of\ false\ negatives}$$

Sensitivities are calculated together with the 95% confidence intervals.

Specificity for pathogen detection will be determined in all samples included in trial. Individual specificities will be calculated for the detection of the respiratory pathogens analyzed by the investigational IVD compared to the standard procedures. Specificity is defined as:

$$Specificity=\frac{number\ of\ true\ negatives}{number\ of\ true\ negatives+number\ of\ false\ positives}$$

Specificities are calculated together with the 95% confidence intervals.

Furthermore, positive and negative predictive agreements shall be calculated.

Genotypic resistance detection by the investigational IVD and phenotypic resistance detection by standard procedures will be evaluated for correlations. Only those data of phenotypic resistances analyzed by standard of diagnostics are included if the respective resistance gene(s) is/are covered by the panel of the investigational IVD. Correlations of the results of the antibiotic resistance detection will be determined between all samples in which resistance detection by standard procedures was performed. Species-specific correlations of antibiotic resistance detection between investigational and standard procedures will be determined by descriptive two-sided 95% confidence intervals. Correlations will be determined by several methods, depending on the relation between the resistance gene(s) identified by the investigational IVD and the phenotypic resistance identified by the standard procedures. Methods include, but are not limited to, sensitivity, specificity, positive and negative predictive agreements.

'Time to Result' (TTR) will be summarized by simple descriptive statistics for investigational and standard procedures separately for each trial center. Only “time to result” of pathogens and resistance genes that are covered by the panel of the

© Copyright 2011 Curetis AG - Confidential

investigational IVD are compared..TTR by pathogen and resistance will be summarized by simple descriptive statistics independently for all individual pathogens and for all individual resistance genes. Times for detection and non-detection respectively are documented per pathogen, resistance gene, and antibiotic resistance. Time for non-detection of pathogens in microbiological culture according to standard procedures.

An interim analysis of the Unyvero test results is planned at up to 1,000 cases. The results of the interim analysis will not be used to modify the protocol or any test procedures.

Missing data will not be imputed.

Discrepant Result Analysis

(Curetis Responsibility)

It is to be expected that due to on average higher analytic sensitivity of nucleic acid amplification methods, pathogens may be detected more frequently by the investigational diagnostic procedure than by culture. Discrepant results of pathogen detection will be evaluated in all samples with different results of the investigational IVD compared to standard methods, except for those samples diagnosed for tuberculosis. Differing results in pathogen detection between the investigational and standard procedure will be calculated and summarized by descriptive statistics. Discrepant results will be managed as indicated in figure 2, see Appendix. Discrepant results will be resolved by sequencing the second fraction of the respective left-over sample. In addition, 10% of the samples with concordant results (i.e. around 200 samples) will also be analyzed by sequencing as a control group.

© Copyright 2011 Curetis AG - Confidential

Appendix

Study Cohort Size Estimation

Pathogen	Occurrence	Lower bound of confidence interval 1	Lower bound of confidence interval 2	Number of positive samples		Cohort size	
				inter val 1	inter val 2	inter val 1	inter val 2
Acinetobacter baumannii	4.1%	90%	80%	73	8	1,780	198
Enterobacter sp.	4.0%	90%	80%	73	8	1,825	203
Escherichia coli	1.6%	90%	80%	73	8	4,562	507
Chlamydophila pneumoniae	2.7%	90%	80%	73	8	2,703	300
Haemophilus influenzae	6.7%	90%	80%	73	8	1,089	121
Legionella pneumophila	1.2%	90%	80%	73	8	6,083	676
Klebsiella pneumoniae	4.0%	90%	80%	73	8	1,825	203
Klebsiella oxytoca	1.0%	90%	80%	73	8	7,299	811
Morganella morganii	2.0%	90%	80%	73	8	3,246	380
Moraxella catarrhalis	0.9%	90%	80%	73	8	8,110	901
Pneumocystis jirovecii	0.6%	90%	80%	73	8	12,165	1352
Proteus sp.	0.8%	90%	80%	73	8	5,122	720
Pseudomonas aeruginosa	23.8%	90%	80%	73	8	307	34
Serratia marcescens	1.7%	90%	80%	73	8	4,294	477
Staphylococcus aureus	21.8%	90%	80%	73	8	335	37
Streptococcus pneumoniae	10.5%	90%	80%	73	8	695	77
Stenotrophomonas maltophilia	3.0%	90%	80%	73	8	2,433	270

Table 4: Estimated Cohort Sizes, calculated for a Sensitivity of 95%.

© Copyright 2011 Curetis AG - Confidential

Table 4: Estimated Cohort Sizes, calculated for a Sensitivity of 95%. .

The minimum number of positive samples n was calculated as:

$$n = 1{,}96^2 \cdot \frac{Sensitivity \cdot (1 - Sensitivity)}{(Sensitivity - 0{,}9)^2}$$ =73, for a lower bound of 90%

and

$$n = 1{,}96^2 \cdot \frac{Sensitivity \cdot (1 - Sensitivity)}{(Sensitivity - 0{,}80)^2}$$ =8, for a lower bound of 80%.

The required sample size (=study size) N was calculated as:

$$N = \frac{n}{k}$$ where k is the frequency of pathogen occurrence.

Discrepant Result Resolution

© Copyright 2011 Curetis AG - Confidential

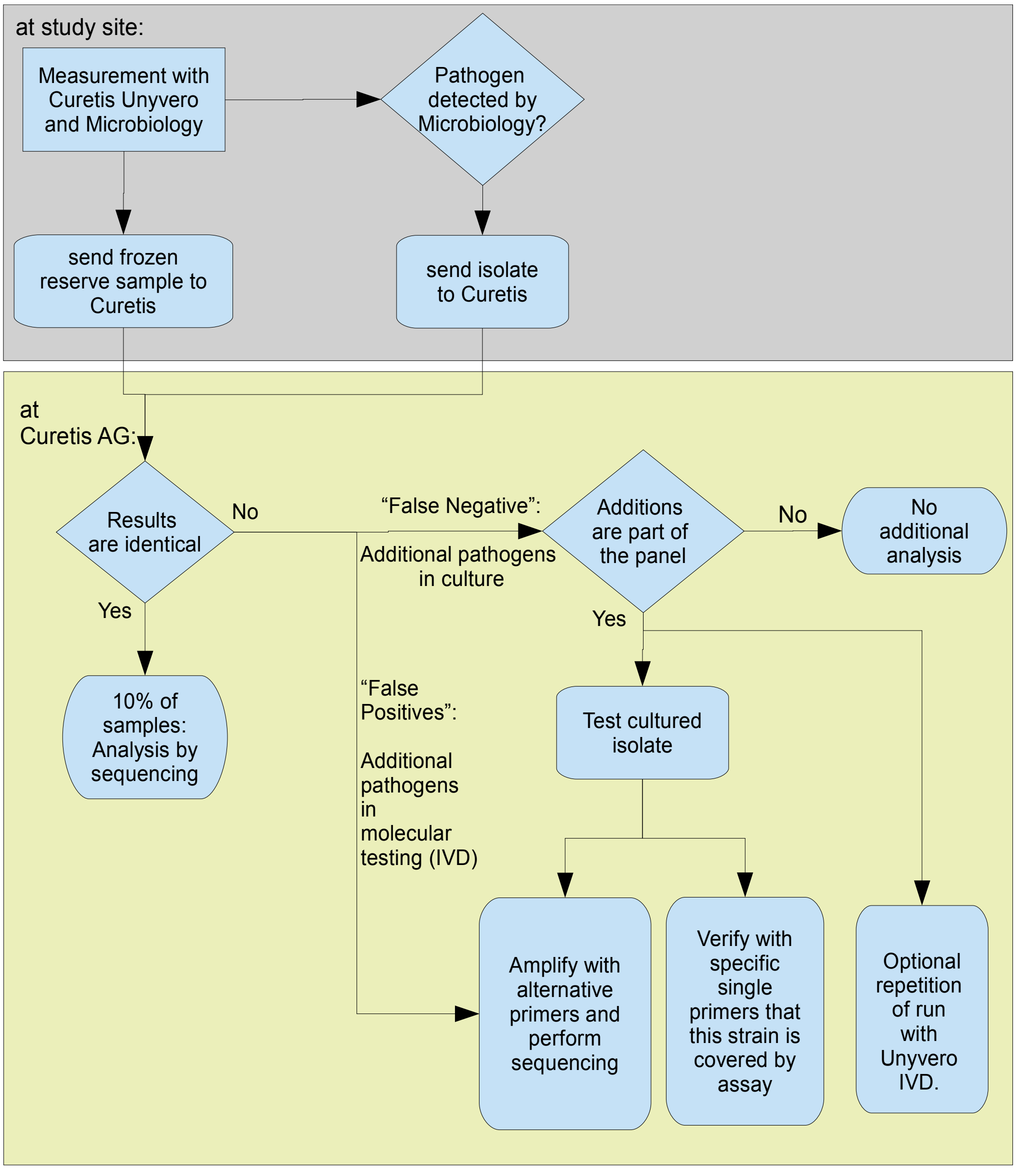

Figure 2: Discrepant Result Resolution.

© Copyright 2011 Curetis AG - Confidential

Literature

- Arias CA et al. (2009) Antibiotic-resistant bugs in the 21st century– a clinical super-challenge. New Engl J Medicine 360;5: 439-443.
- Höffken et al. (2009) Epidemiology, diagnosis, antimicrobial therapy and management of community-acquired pneumonia and lower respiratory tract infections in adults. Guidelines of the Paul-Ehrlich-Society for Chemotherapy, the German Respiratory Society, the German Society for Infectiology and the Competence Network CAPNETZ Germany. Pneumologie 63(10):e1-68
- Kollef M et al. (1999) Inadequate antimicrobial treatment of infections. Chest 115: 462-474.
- Lorenz et al. (2003) Nosocomial pneumonia: prevention, diagnosis, treatment. Pneumologie. 57(9):532-45.
- Luna CM et al. (1997) Impact of BAL data on the therapy and outcome of ventilator-associated pneumonia.Chest 111(3):676-85.
- Rello J et al. (1997) The value of routine microbial investigation in ventilation-associated pneumonia. Am J Resp Crit Care Medicine. Vol 156: 196-200.

Document-related SOPs

The following SOPs will be provided until start of study:

- SOP 'Unyvero Reproducibility Study'
- SOP 'Discrepant Result Resolution'
- SOP 'Quality Control Runs'

- SOP 'Handling of Isolates'
- SOP 'Return Shipments'

Glossary

Serious adverse device effect (SADE)
A serious adverse device effect is defined as an adverse device event that has resulted in any of the consequences characteristic of a serious adverse event or that might have led to any of these consequences if suitable action had not been taken or intervention had not been made or if circumstances had been less opportune which is related to the device, it's use or attendant instructions or label.

Unexpected adverse device effect (UADE)
An unexpected adverse device effect is an adverse device effect related to any untoward and unintended response to a medical device which is not anticipated by the protocol and attendant documentation, such as the Investigator Brochure.#

'True' Positive (Negative) Samples
True positive (negative) samples are defined as positive (negative) in microbiology standard method and positive (negative) in the Unyvero Pneumonia Application..
'False' positive samples are positive samples in the Unyvero Application, but negative in the microbiology standard test. 'False' negative samples are negative in

© Copyright 2011 Curetis AG - Confidential

the Unyvero test, but positive in microbiology standard test (see table 5).

		Standard-of-Care Result +	Standard-of-Care Result -
Unyvero Application Result	+	True positive	False positive
	-	False negative	True negative

Table 5: Definition of True or False Positive / Negative Samples

IVD: in-vitro diagnostic device
HIS: Hospital information system
LIS: Laboratory information system
SOP: Standard operating procedure
TTR: Time to Result

© Copyright 2011 Curetis AG - Confidential

Curetis AG
Max-Eyth-Straße 42
71088 Holzgerlingen
Germany

Tel: +49(0)7031 49195-10
Fax: +49(0)7031 49195-19

www.curetis.com
