# Supplementary material for: Detection of Pneumonia Associated Pathogens Using a Prototype Multiplexed Pneumonia Test in Hospitalized Patients with Severe Pneumonia
Source: PLoS One. 2014 Nov 14;9(11):e110566. doi: 10.1371/journal.pone.0110566 (PMC4232251; doi:10.1371/journal.pone.0110566)
Supplement: Study Registration S1 — Application for registration for trial DRKS00005684. (PDF) [file pone.0110566.s004.pdf]

# Antrag zur Registrierung der Studie DRKS00005684

datenmanagement@drks.de
**Gesendet:** Montag, 27. Januar 2014 18:18
**An:** Keller, Peter

Sehr geehrter Herr Dr. med. Keller,

Vielen Dank, dass Sie die Daten Ihrer Studie "Detektion von Lungenentzündungskrankheitserregern und Antibiotika-Resistenz-Genen mit Hilfe der Curetis Unyvero® Pneumonia Application" an das DRKS übermittelt haben. Diese werden nun einer Prüfung unterzogen. Gegebenenfalls werden Sie in Kürze eine E-Mail zur Klärung von Rückfragen erhalten.
Beachten Sie bitte, dass Ihr Datensatz auf der öffentlichen Seite des DRKS erst erscheint und Ihre Studie erst dann als registriert gilt, wenn ein vollständiger und valider WHO-Datensatz vorliegt. (vgl. WHO Trial Registration Data Set - http://www.who.int/ictrp/network/trds/en/index.html)

Vielen Dank für Ihre Mitarbeit.

Mit freundlichen Grüßen
Ihr DRKS-Team

---

**Your application for registration for trial DRKS00005684**

Dear Dr. med. Keller,

Thank you for passing your trial "Detection of Pneumonia associated Pathogens and Antibiotic Resistance Genes using the Curetis Unyvero® Pneumonia Application" on to the DRKS. It will be reviewed for integrity and plausibility by a data manager. If necessary you will receive an e-mail with the demand to review check results and queries.
Please note that a minimum amount of trial information must be entered correctly and completely for a given trial before it can be considered fully registered (see WHO Trial Registration Data Set - http://www.who.int/ictrp/network/trds/en/index.html).
After registration your trial will be displayed on the public web site.

Thank you for your cooperation.
Please do not hesitate to contact us.

Kind regards
DRKS-team
